# Horizontal biases in rats’ use of three-dimensional space

**DOI:** 10.1016/j.bbr.2011.02.035

**Published:** 2011-09-23

**Authors:** Aleksandar Jovalekic, Robin Hayman, Natalia Becares, Harry Reid, George Thomas, Jonathan Wilson, Kate Jeffery

**Affiliations:** a Institute of Behavioural Neuroscience, Department of Cognitive, Perceptual and Brain Sciences, Division of Psychology and Language Sciences, University College London, 26 Bedford Way, London WC1H 0AP, UK; b Axona Ltd, Unit 4U, Long Spring, Porters Wood, St. Albans AL3 6EN, UK

**Keywords:** Navigation, 3D, Vertical, Foraging, Detour, Rats

## Abstract

Rodent spatial cognition studies allow links to be made between neural and behavioural phenomena, and much is now known about the encoding and use of horizontal space. However, the real world is three dimensional, providing cognitive challenges that have yet to be explored. Motivated by neural findings suggesting weaker encoding of vertical than horizontal space, we examined whether rats show a similar behavioural anisotropy when distributing their time freely between vertical and horizontal movements. We found that in two- or three-dimensional environments with a vertical dimension, rats showed a prioritization of horizontal over vertical movements in both foraging and detour tasks. In the foraging tasks, the animals executed more horizontal than vertical movements and adopted a “layer strategy” in which food was collected from one horizontal level before moving to the next. In the detour tasks, rats preferred the routes that allowed them to execute the horizontal leg first. We suggest three possible reasons for this behavioural bias. First, as suggested by Grobety and Schenk [5], it allows minimisation of energy expenditure, inasmuch as costly vertical movements are minimised. Second, it may be a manifestation of the temporal discounting of effort, in which animals value delayed effort as less costly than immediate effort. Finally, it may be that at the neural level rats encode the vertical dimension less precisely, and thus prefer to bias their movements in the more accurately encoded horizontal dimension. We suggest that all three factors are related, and all play a part.

## Highlights

► Rat spatial behaviour was compared between horizontal and vertical dimensions. ► In both foraging and detour tasks, rats preferred horizontal over vertical movements. ► Rats also preferred routes where the horizontal leg occurred first rather than last. ► A horizontal bias is energetically adaptive and may reflect a parallel encoding bias. ► The preference for horizontal-first routes may reflect temporal effort discounting.

## 1 Introduction

Spatial cognition in rats has long been a model system with which to explore links between neural encoding of the world and behaviour. While navigation strategies and coding processes in two-dimensional environments have been extensively studied, relatively little is known about how animals and humans encode and navigate in three-dimensional (3D) spaces. However, the world is three dimensional, with an often complex topography. Even rats, which are mostly surface-travelling, live in branching subterranean 3D burrow systems [3] and may need to encode, store and retrieve spatial representations that include vertical information. Many animals also move freely above ground in all three dimensions, either by swimming, flying or gliding, or else by climbing through lattice structures such as trees. Understanding real-world spatial representation therefore requires an understanding of how animals encode and use information about the vertical dimension.

The purpose of the present study was to address this question by studying how rats approached two navigational tasks, foraging and detouring, in two environments extending into the vertical dimension. Foraging and detouring are self-organised, naturalistic behaviours that have not been previously studied in three dimensions. Our study was motivated by neurobiological findings suggesting a weak or absent metric encoding of vertical space [6], together with prior observations that rats and other animals separate the vertical and horizontal components of navigation [5,8], and also theoretical considerations that suggest that complete volumetric encoding of 3D space would be computationally extremely costly [10].

Relatively little prior work has explored the 3D encoding of space in animals. It has previously been shown that the presence of terrain slope, which provides an additional source of orienting information, facilitates learning and navigation accuracy in 2D environments in humans [16], rodents [12] and pigeons [13]. Such behavioural findings are in line with the effects of terrain slope on the firing properties of hippocampal place cells, which encode distinct locations within horizontal environments [14]: Jeffery et al. [9] showed that place cells can use information about tilted surfaces to orient in a symmetric 2D space, although they do not seem to show true 3D encoding [11].

Navigation studies in volumetric 3D environments have also shown that vertical and horizontal spaces might be used and encoded differentially. Grobety and Schenk [5] were among the first to pioneer the study of animal navigation in 3D environments, using a cubic lattice maze which allowed rats to move in all three dimensions with relative ease. They showed that in a goal-directed navigation task rats had strong movement preferences in the horizontal dimension, and that learning in such environments might involve a two-step process: first, identifying the vertical coordinate, followed by second, navigating to the horizontal place. Comparable studies with the blind Mexican cave fish, *Astyanax fasciatus*, in three-dimensional volumetric space have provided evidence for strong preferences for vertical information in these fish [8]. Similar results were obtained with humans while navigating through a building [7]. Here, experienced participants preferred to use a strategy in which they headed towards the vertical position of the goal first, in order to navigate subsequently towards its horizontal position. However, in contrast to these results, another human study has shown that humans determined the position of an object in the horizontal dimension first, before moving vertically between floors [2]. Thus, there is some disagreement about the degree to which the vertical dimension is preferentially encoded by animals moving in 3D space.

**Fig. 1:**
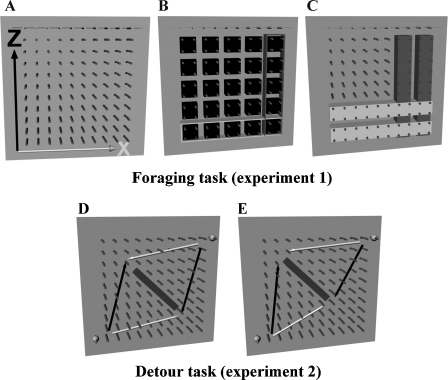
Schematic of the pegboard experiments (Experiments 1 and 2). (A–C) The foraging setup (Experiment 1), (D and E) the detour setup (Experiment 2). (A) The pegboard, a climbing wall with 100 pegs attached to a vertical wooden board (side length 121 cm × 121 cm). (B) 25 reward regions highlighted (black boxes), each region consisting of four pegs. One of the four pegs in each region was rewarded, with the reward peg changing randomly between trials. (C) Five horizontally adjacent regions form a layer (two layers shown in white) and ﬁve vertically adjacent regions form a column (two columns shown in grey). (D) The detour setup (Experiment 2). The pegboard is shown with 102 pegs attached, and the inserted barrier as it was initially positioned during detour testing. The 2 grey spheres indicate start and goal positions. The white arrows show the shallow-ﬁrst legs of the two possible routes, both outward and return, and the black arrows show the steep-ﬁrst legs. (E) Asymmetrically placed barrier.

**Fig. 2:**
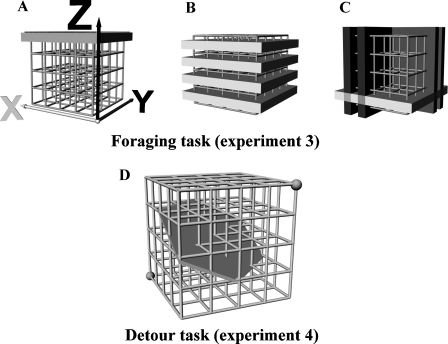
Schematic of the lattice maze experiments. (A–C) The foraging setup (Experiment 3). (D) The detour setup (Experiment 4). (A) The lattice maze (side length 50 cm × 50 cm × 50 cm), a climbing cube constructed out of 64 smaller cubes. (B) The maze consists of 4 layers (white), each of the layers being baited at 6 positions, which varied randomly between trials. (C) Shown are examples of layers (white) and slices (grey and black), which were assigned for the layer-by-layer analysis and the slice-by-slice analysis. (D) The detour setup with inserted solid barrier.

We aimed to confirm Grobety and Schenk's results [5] and extend them to foraging and detour tasks, to explore how rats allocate their time when able to choose freely between horizontal and vertical movements. Both kinds of task were conducted in two kinds of apparatus, the “pegboard” (Fig. 1) which is quasi-two dimensional, with one vertical and one horizontal dimension, and the “lattice maze” (Fig. 2) which is three-dimensional. Table 1 shows the design of the experiment. In the foraging task, rats were free to explore the apparatus and retrieve food from multiple locations scattered throughout the space. Their movements were analysed with respect to how horizontal and vertical transitions were distributed and how far their paths deviated from the shortest path. In the detour task, rats were trained to shuttle back and forth between two positions, one low and one high, before being presented with a barrier around which they needed to detour. Rats could thus choose between detour paths for which the initial segments were initially horizontal vs. initially vertical. In both types of task, the distribution between horizontal and vertical movements was compared. As we will show, rats used horizontal and vertical space differently, showing a preference for horizontal movements and strategies and also, unexpectedly, a marked preference for deferring the vertical component of the journey. We present these results, below, and discuss them in terms of optimisation and encoding constraints.

## 2 Materials and methods

### 2.1 Subjects

20 male Lister hooded rats, weighing 310–440 g and 2–9 months of age, were used throughout all experiments. Animals were housed singly or in pairs in Perspex cages. Animals had ad libitum water access and restricted feed, in which food was available to maintain 90% of the free-feeding weight. Lights were on from 8 a.m. until 7 p.m. and off between 8 p.m. and 7 a.m. Dawn and dusk were simulated with lights on half strength between 7 a.m. and 8 a.m. and 7 p.m. and 8 p.m., respectively. The temperature in the holding room was kept constant at 21 ± 2 °C. All procedures carried out during these experiments were licensed by the UK Home Office, subject to the restrictions and provisions contained in the Animals (Scientific Procedures) Act of 1986.

### 2.2 Apparatus and testing room

All experiments were conducted in the same room under dimmed light conditions. The room contained electronic equipment and shelves which were visible throughout the experiments.

### 2.3 Pegboard

The pegboard arena (Fig. 1) consisted of a wooden board (121 cm × 121 cm square), with evenly spaced holes (0.9 cm diameter and 10 cm spacing). Pegs (wooden dowels 17 cm × 0.9 cm) were plugged into the holes to provide footholds for the rats to climb on. The pegboard was placed in a vertical position, with the lower edge 80 cm above ground, throughout the experiments. The board was cleaned and pegs were interchanged after each set of trials in order to remove local olfactory information. A 2D Cartesian coordinate system, with a horizontal (*X*) and a vertical (*Z*) axis (as shown in Fig. 1A), was defined to describe spatial positions.

### 2.4 Lattice maze

The lattice maze (shown in Fig. 2) was built using construction materials from a children's toy-set (Quadro, Hamburg, D). Hollow cubes were created by attaching tubes (length: 12 cm, diameter: 1 cm) to connector pieces (diameter: 3 cm). These cubes were then assembled into a 4 × 4 × 4 cubic maze (50 cm × 50 cm × 50 cm). The maze was raised 50 cm above the ground, supported on a wooden frame; it thus had no floor surface. The top of the maze was covered with a wire mesh cover, thus preventing rats from climbing onto the top of the maze. Following construction, the maze was painted with polyurethane non-slip coating (Protecta Kote, New Venture Products Ltd, Wantage, UK), which provided better grip, and prevented rats from slipping. To describe positions in space, a 3D Cartesian coordinate system was defined with the three perpendicular axes being aligned with the lattice maze. The origin was defined as the front lower left corner of the maze, as shown in Fig. 2A, with the *X* and *Y* axes being the two horizontal axes and the *Z*-axis being aligned vertically. The lattice maze was randomly rotated and cleaned daily in order to extinguish the possibility that rats use odour cues in order to navigate through the maze.

Experiments 1 and 2 were run in reverse order, with the lattice maze experiment first, but are presented in order of increasing dimensionality of the apparatus, for clarity.

### 2.5 Experiment 1: foraging on the pegboard

This experiment was specifically designed to prevent stereotypic behaviour and to observe rats’ foraging abilities under more naturalistic conditions. We hypothesised that rats will show strong horizontal movement biases and apply a horizontally biased layer-by-layer strategy to retrieve food in such environments.

#### 2.5.1 Subjects

Subjects were eight male Lister Hooded rats which were previously used in Experiment 2 and which therefore already had good climbing abilities. Rats were aged 6–7 months at the beginning of the experiments, and weighed 330–440 g.

#### 2.5.2 Experimental procedure

100 pegs (wooden dowels 17 cm × 0.9 cm) were inserted into the pegboard with a peg-to-peg distance of 10 cm (as shown in Fig. 1A). Malt-flavoured rice was semi-randomly scattered on the pegs, with two rice grains per position (25 positions in total). Groups of four pegs, each in a 2 × 2 array, were used to outline 25 separate reward regions (Fig. 1B) with food in all 25 regions but the exact location varying between the four pegs in each region, across trials. This protocol was chosen for easier recording and to allow a simple quantification of foraging strategies. Starting positions were kept constant throughout the whole experiment, with two of the rats starting from the bottom left, two from the bottom right, two from the top left and two from the top right. A trial began when the animal was placed on the apparatus facing towards one of the four corners and was finished after an animal collected all food positions or was manually removed by the experimenter after 6 min. Experiments were conducted for ten days, with one session per rat and day.

Rats’ positions were determined using DacqTrack (Axona, St. Albans, UK), which tracked the black coloured head against the pale background.

#### 2.5.3 Analysis

Positions were analysed using custom-written Matlab programmes (R2008b, The Mathworks). Data were smoothed and positions were arranged in 5 × 5 bins, with each bin representing one reward region consisting of four pegs (Fig. 1B). The order of retrieved food positions was marked manually on a separate sheet by a second experimenter. Bin crossings were calculated and used to compare horizontal and vertical movements.

#### 2.5.4 Ordinal distance analysis

The ordinal distance analysis was undertaken to try and discover regularities in the food retrieval pattern on a given trial. It was used to investigate the spatial distribution of food collection specifically, and not activity *per se*. The hypothesis under consideration was that rats would tend to retrieve all the food from a given horizontal layer before ascending or descending to a different layer. Each food location was therefore given an ordinal number from 1 to 25, corresponding to where in the complete set of 25 visits that location was visited on that trial. If the “layer strategy” hypothesis was correct, then the ordinal numbers from that layer would tend to cluster together and those from different layers would be more widely distributed. By contrast, if the comparison were made for vertical strips (“columns”) rather than layers then there should be no such clustering. Thus, the “ordinal distance” (difference between ordinal numbers) was computed for every pair of food locations, and then compared for pairs that were in the same layer, or column, vs. different layers or columns.

To accomplish this analysis, the pegboard was divided into layers and columns and ordinal distances (OD) were calculated as shown in (1).

(1) $$OD_{k}=\frac{\sum_{i=1}^{n-1}\sum_{j=i+1}^{n}\left|x_{i}-x_{j}\right|}{n(n-1)/2}$$

The absolute difference of all possible ordinal number pairs (|*x*_*i*_ − *x*_*j*_|: ordinal distance pair for events *i* and *j*) per layer or column was calculated and summed up separately for each layer and column with $\sum_{i=1}^{n-1}\sum_{j=2}^{n}\left|x_{i}-x_{j}\right|$ and was divided by the number of possible pairs *n*(*n* − 1)/2 (whereas n equals the amount of retrieved food positions per layer or column, and *n* ≥ 2). Subsequently the average OD of all layers and all columns was calculated, whereby *p* represents the number of layers or columns with two or more ordinal numbers (shown in (2)).

(2) $$OD=\frac{\sum_{k=1}^{p}OD_{k}}{p}$$

#### 2.5.5 Genetic algorithm and ordinal distances for an optimal path

To compare actual rat data with values that would arise from a completely ideal route (“ideal” in purely distance terms, assuming no distinction between vertical and horizontal), optimal paths for each trial were calculated using the same start positions and the same fixed retrieval positions as in the experiment itself. The first retrieval position was defined as the food position adjacent to the start on the *X* axis.

These optimisations were calculated using custom-written Matlab (R2008b, The Mathworks) programs (based on previous code by Joseph Kirk, 2009). Standard genetic algorithm procedures were applied [15], by first creating a matrix of 100 random routes (initialisation) and then optimising these orders by selecting the best one out of a random selection of four routes (natural selection) and rejecting the other three. From each of the best 25 routes, three new routes were created by manipulating the route order via swapping, flipping and sliding of random positions (mutation). This algorithm was run 10000 times with the best route being stored. In order to prevent local minima solutions and cover multiple best solutions the whole process was executed 1000 times and the shortest routes were evaluated further using the ordinal distance calculation as described earlier. Control ordinal distances were calculated using these data sets as shown in (1). As optimised routes were generated only based on distance properties in an otherwise isotropic environment, no specific travelling biases were expected using such an approach. Thus, we could compare actual rat performance in a normal environment having gravity against optimal performance in a theoretical, gravity-free environment.

#### 2.5.6 Ordinal distance ratios

Ordinal distance ratios were calculated by dividing observed ordinal distances with the optimised ordinal distances generated by the genetic algorithm. A ratio around 1 for the layer-by-layer analysis therefore would indicate that the rats had applied a random strategy, ignoring the dimensions (horizontal vs. vertical), as the ordinal distances of observed trials would equal the ordinal distances of optimised data. Smaller values within a layer would indicate clustering of choices close to each other within that layer and therefore indicate that animals would be biased towards adopting a layer strategy, and higher values would indicate that animals used a vertically biased strategy (which would seem unlikely *a priori*). Likewise ratios smaller than 1, obtained by a column-by-column analysis would imply that rats were biased towards the vertical dimension and, conversely, values bigger than 1 would indicate that rats were horizontally biased.

#### 2.5.7 Movement analysis

As with the rat data, bin crossings were calculated for the optimised data sets and used to compare horizontal and vertical movements.

#### 2.5.8 Statistics

All comparisons were made using ANOVA unless otherwise stated.

### 2.6 Experiment 2: detour task on the pegboard

This detour experiment tested whether rats can dynamically re-programme a navigational path in the vertical dimension, as they can in the horizontal, and to explore how they would distribute their journey between vertical and horizontal components.

#### 2.6.1 Subjects

Six male naive male Lister Hooded rats weighing 310–360 g during the experiment and 2 months old at the beginning of the experiment, were used in this study.

#### 2.6.2 Tracking

Rats were tracked in this setup with DacqUSB (Axona, St. Albans). Retro-reflection foil (3 M, Neuss, D) was attached symmetrically on either side of the rats’ torso. A light source was placed next to the camera, allowing accurate tracking as the reflected light was returned directly to the camera, which was positioned next to the light source. Positions were analysed using custom-written Matlab programmes (R2008b, The Mathworks).

#### 2.6.3 Experimental procedure

The pegboard, configured as shown in Fig. 1D, was used for this study, with three white plastic walls added to the left and right side as well as the top. During the first week rats were placed on the pegboard in pairs, to become familiar with the environment and acquire good climbing skills. CocoPops (® Kellogg Company) were used as reward during the entire training and testing period. All rats were placed singly at the start location (bottom-left corner) for training and testing (see Fig. 1D). A trial started after the animal collected the first food reward at the start position and finished after it had travelled from the start position to the goal and back, collecting food at both locations. The trial was manually stopped by the experimenter after 6 min if the rat did not finish the task. Training was conducted in sets of 10 consecutive trials within a day and continued until rats directly navigated from the start to the goal and back again. The acceptance criterion to enter the testing stage was that the first three consecutive trials were direct paths to the start and goal positions.

#### 2.6.4 Initial symmetrical barrier

Once rats had achieved a satisfactory performance level, an opaque barrier (48 cm length, as shown in Fig. 1D) was introduced into the maze, midway between the start and the goal. The effect of this was to create two possible two-legged detours of equal lengths, but different initial legs; one steep with a strong vertical component and the other one shallow with a strong horizontal component, allowing testing for pre-existing preferences. Each rat undertook one set of 10 barrier trials. At this stage, as well as recording the route taken, path length and path time, the path choice was manually recorded by the experimenter, for both the outbound and the inbound routes.

#### 2.6.5 Asymmetrically placed barrier

As we will show in the results, rats preferred detours in which the horizontal component occurred first. In order to study whether these horizontal biases were absolute or traded off against distance or effort, in some test trials the barrier was offset either up and to the left or down and to the right (Fig. 1E). Before starting the offset condition, rats completed three trials without any barrier in order to verify that they still were able to navigate straight towards the goal. To prevent stereotypical behaviours during testing, barriers were presented in a random order in either an upper, middle or lower location, each of which was presented 10 times, forming an entire session with 30 trials.

#### 2.6.6 Analysis

Decision frequencies were analysed for all conditions.

### 2.7 Experiment 3: foraging on the lattice maze

As on the pegboard, this experiment was designed to study rats’ foraging behaviour in 3D environments. Stereotypic behaviours were avoided by using a semi-random design with changing food positions. As in Experiment 1, we hypothesised that rats would show strong horizontal movement biases and that foraging would be horizontally biased.

#### 2.7.1 Subjects

Eight male naive Lister Hooded rats, aged 2–3 months at the beginning of the experiments and weighing 250–300 g throughout the procedure, were used.

#### 2.7.2 Tracking

Rats’ horizontal positions were tracked from an overhead camera using DacqUSB (Axona, St. Albans, UK). A light-source was placed next to the camera emitting light parallel to the direction of view of the camera. Retro-reflection foil (3 M, Neuss, D) was attached to each rat's head, as in Experiment 2. Vertical positions were determined using key presses made by an observing experimenter, indicating the current layer position of the rat. Positions were reconstructed by calibrating the outer positions of each layer prior to the experiment. Paths and bins were rescaled accordingly. Data were smoothed with Tint (Axona, St. Albans, UK) and positions were reconstructed in 3D with custom-written Matlab programmes (R2008b, The Mathworks). Positions were calculated for the four layers, which were divided into 4 × 4 bins, with each bin thus representing one cube of the lattice. The order of retrieved food positions was marked on a separate sheet by a second experimenter. In order to match horizontal and vertical movements, the former were down-sampled into bins and cube crossings were compared.

#### 2.7.3 Experimental procedure

All rats received 15 min of handling per day over the course of three days. Following this, 10 days of 30 min of training were experienced by each rat. Pre-training was undertaken to allow rats to become familiar with the lattice maze and to acquire adequate motor skills. One week of testing followed this procedure, in which two trials were carried out per rat per day. Rats had to forage for 24 pseudo-randomly placed food positions within the maze. Two honey-flavoured rice grains were placed on each baited position (intersection points in the lattice), with 6 out of 25 possible positions per layer. Start positions were kept constant during testing with four rats starting from the bottom corner and four rats starting from the top. A trial was finished after an animal collected all food positions or was discontinued by the experimenter after 6 min. Experiments were conducted for five days, with one session per rat and day.

#### 2.7.4 Analysis

For analysis, the maze was divided into slices either horizontally in the *X*–*Y* plane (termed “layers”; Fig. 2B), or vertically in either the *X*–*Z* or *Y*–*Z* planes (“slices”; Fig. 2C).

To compute movements in the *X*, *Y* and *Z* dimensions, the lattice maze was divided into 64 hollow cubes, with each of the four layers consisting of a 4 × 4 array of 16 cubes. Cube crossings were calculated for each dimension and used to compare horizontal and vertical movements.

Ordinal distances were calculated as described in Experiment 1 (see Section 2.5), with this measure being applied to a layer-by-layer analysis and to the two orthogonal slice-by-slice analyses, as shown in Fig. 2C. The same approach as in Experiment 1 (see Section 2.5) was chosen to calculate the ordinal distance ratios.

Optimal routes in 3D were calculated as in Experiment 1 by using a genetic algorithm to optimise path lengths (see Section 2.5). Cube crossings were obtained for the optimised data sets and were used to evaluate horizontal and vertical movements.

### 2.8 Experiment 4: detour task on the lattice maze

As in Experiment 2, this detour experiment was conducted to determine whether rats showed systematic path preferences whilst navigating on the lattice maze. After having learned to shuttle back and forth between food locations, a solid barrier was introduced, to force them to execute a detour. This allowed rats to take either a vertical-first or vertical-last route.

#### 2.8.1 Subjects

Six naive male Lister Hooded rats were used in this experiment. They were 2 months old at the beginning of the experiment and weighed 311–379 g throughout the experiment.

#### 2.8.2 Behavioural measurements

The experimenters recorded level transitions and times to travel between two locations. During the detour phase, following insertion of the solid barrier (as shown in Fig. 2D) path were recorded manually.

#### 2.8.3 Experimental procedure

Rats were trained to shuttle between a start and a goal location, with half of the rats starting from the top and half starting from the bottom. The animals were familiarised with the maze one week prior to training, allowing all rats to acquire good climbing abilities. The training phase lasted for 15 days, with one training session per day, consisting of 10 trials. A trial was finished after an animal travelled from the start position to the goal and back and collected the food. The trial was manually stopped by the experimenter if the rat did not finish the task within 6 min. During the testing phase a solid barrier (shown in Fig. 2D) was introduced to determine which paths rats chose to reach the goal. All decisions were recorded by the experimenter manually.

#### 2.8.4 Analysis

Time taken, vertical cube crossings of the training period were analysed as well as the obtained frequencies in the solid barrier and 16-hole barrier trial.

## 3 Results

### 3.1 Experiment 1: foraging on the pegboard

**Fig. 3:**
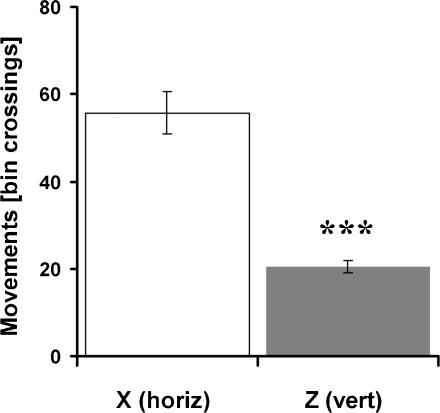
Experiment 1—movement behaviours during foraging on the pegboard. Bin crossings in the horizontal (X) and vertical (Z) dimension. Pooled data: *n* = 8 rats, 10 days, 2 trials per day and rat.

Comparison of horizontal and vertical movements revealed a significant difference, with rats exhibiting more horizontal movements (paired *t*-test: *t*_9_ = 9.46, *P* < 0.001, Fig. 3).

To test whether these movement biases were a consequence of the experimental setup the same measurements were applied to the control data sets obtained with a genetic optimisation algorithm. Horizontal (*X*: 8.57 ± 0.17) and vertical (*Z*: 8.38 ± 0.26) movements of these sets did not show any significant differences (paired *t*-test: *t*_9_ = 0.49, *P* > 0.2), thus revealing that movement biases were not a general effect of the apparatus.

**Fig. 4:**
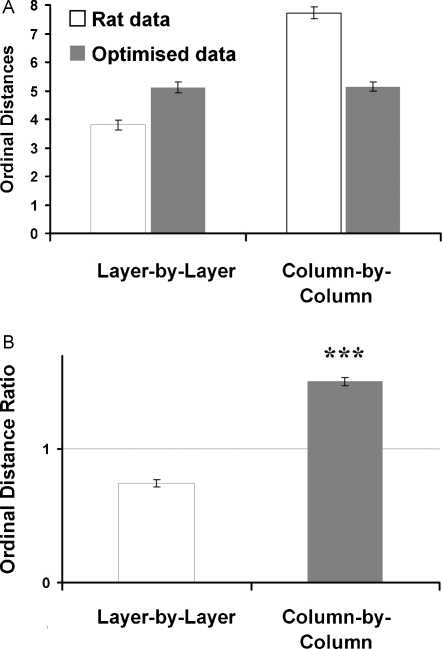
Experiment 1—the ordinal distances in (A) and ordinal distance ratios in (B) of the foraging experiment on the pegboard. The ordinal distance analysis was undertaken to underpin regularities in the food retrieval pattern on a given trial. Ordinal distance ratios were calculated by dividing ordinal distances of rats with ordinal distances of optimised paths. Smaller values in the layer analysis indicate clustering of choices within layers and therefore indicate that animals would be biased towards adopting a layer strategy, and higher values would indicate that animals used a vertically biased strategy. Pooled data: *n* = 8 rats, 10 days, 2 trials per day and rat.

The ordinal distance analysis (as described in Section 2) was applied to the data obtained from the actual experiment and was compared against optimised paths as determined using the genetic algorithm (shown Fig. 4A). For real data, the ordinal distance for layers was 3.8 ± 0.17 and for columns was 7.73 ± 0.21; for the control data, the distance for layers was 5.12 ± 0.2 and for columns was 5.14 ± 0.15. A two-factor ANOVA revealed significant main effects of data type (real data vs. optimised data, *F*_1,28_ = 12.09, *P* < 0.01), a significant main effect of the dimension (layer vs. slice, *F*_1,28_ = 116.05, *P* < 0.001) and a significant interaction of data type and dimension (data type × dimension, *F*_1,28_ = 113.86, *P* < 0.001).

In order to underpin these results, ordinal distance ratios were calculated by dividing the ordinal distance for each rat with the ordinal distance of the optimised data, and then averaged. The ordinal distance ratios of a layer-by-layer analysis were 0.75 ± 0.03, which was significantly smaller than the ordinal distance ratios of a column-by-column analysis, which was 1.51 ± 0.03 (paired *t*-test: *t*_7_ = 20.377, *P* < 0.001, Fig. 4B). Thus, the ordinal distance analyses revealed that rats used a horizontally organised layer-by-layer strategy, in which food items located within a given horizontal layer tended to be collected together significantly more often either than in slices, or in the artificially created “optimised” paths.

### 3.2 Experiment 2: detour task on the pegboard

Rats were trained in this task for 3–10 days, until they reached a satisfactory navigation level, indicated by direct paths towards the goal locations.

**Fig. 5:**
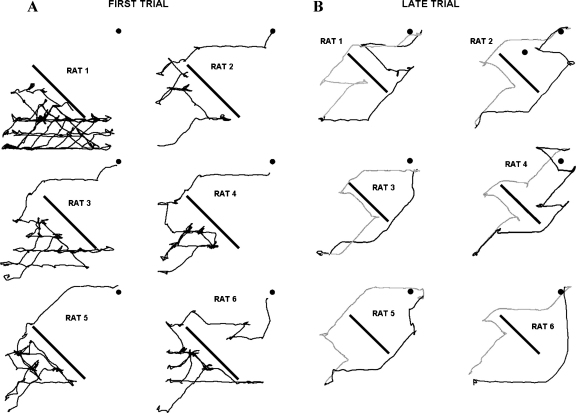
Experiment 2—path choices of all six animals on the pegboard during (A) ﬁrst insertion of a symmetrical barrier and (B) representative paths of all animals during later trials. Upward paths are shown in black and downward paths in grey.

**Fig. 6:**
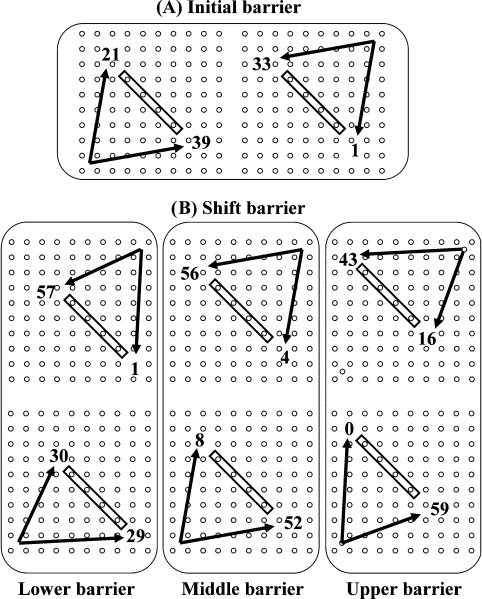
Experiment 2—pooled data of path choices on the pegboard during insertion of symmetrical and asymmetrical barriers. Results of the initial condition are shown in (A), whereby frequencies for shallow and steep paths are indicated for both upward and downward navigation. (B) The frequencies obtained during placement of an asymmetrical barrier. Pooled data: *n* = 6 rats, 10 trials per rat in initial and follow-up condition, 30 trials (10 lower, 10middle, 10 upper) per rat in asymmetrical condition.

Insertion of a symmetrically placed barrier forced rats to detour around the obstacle, taking either a vertical-first or vertical-last route. Interestingly, the first trial differed from all the others: all rats used the vertical-first route in the first trial when navigating upwards (Fig. 5A). This may have been a novelty or anxiety response: it was observed that rats showed a strong reaction to the change during the first barrier trials, whereby they navigated towards the barrier and explored it intensely and frequently travelled back to the start point to check for food. Thereafter we observed horizontal biases in the detour patterns, reflected in strong preferences for initially horizontal paths (examples shown in Fig. 5B; pooled data in Fig. 6). This effect was significant in the inbound journeys (33 initially horizontal paths out of 34 completed trials, one-sample *t*-test, *t*_5_ = 17, *P* < 0.001). On the outbound journeys initially horizontal paths were chosen more often, yet not significantly (39 initially horizontal paths out of 60 completed trials, one-sample *t*-test, *t*_5_ = 1.47, *P* > 0.05).

After these initial trials the same barrier was randomly presented in one of two counterbalanced asymmetrical positions. The usual symmetrical position was also presented again, to establish baseline performance. As in the first set of barrier trials, symmetrical placement led to a significant usage of initially horizontal routes (shown in Fig. 6A), both when navigating upwards and downwards (inbound journey: 52 initially horizontal paths out of 60 completed trials (52/60), test, *t*_5_ = 6.57, *P* = 0.001, outbound journey: 56 initially horizontal paths out of 60 completed trials (56/60), test, *t*_5_ = 9.69, *P* < 0.001). Asymmetric placement of the barrier (shown in Fig. 6B) introduced a longer more indirect vs. a shorter and more direct route to reach the goal, and produced a significant effect on route choices. For displacement of the barrier upwards and to the left, in which the previously preferred horizontal-first route was now also the shorter/more direct, the choices shifted from 52/60 (symmetrical barrier) to 59/59 (Linear mixed model, estimates of fixed effects, *t*_18_ = 1.49, *P* = 0.15). On the return route, choices shifted from 56/60 to 43/59 (Linear mixed model, estimates of fixed effects, *t*_18_ = 4.9, *P* < 0.001). For displacement of the barrier downwards and to the right, where the previously preferred route was now longer and less direct, the outgoing preferences shifted from 52/60 to 29/59 horizontal-route choices (Linear mixed model, estimates of fixed effects, *t*_18_ = 2.533, *P* < 0.05). On return, choices shifted from 56/60 to 57/58 (Linear mixed model, estimates of fixed effects, *t*_18_ = 0.2, *P* > 0.05).

In summary, rats showed a strong bias to use paths with strong horizontal initial segments. This bias was relative rather than absolute, however, because it could be altered by increasing the “cost” of taking the horizontal-first route.

### 3.3 Experiment 3: foraging on the lattice maze

**Fig. 7:**
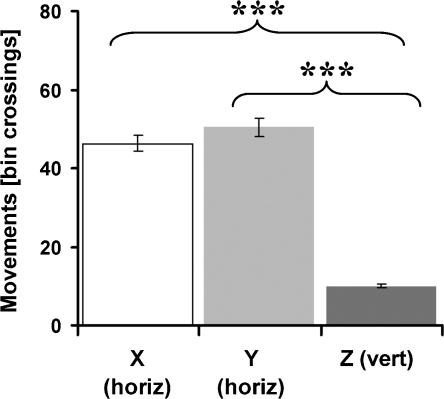
Experiment 3—bin crossings in the horizontal (*X* and *Y*) and vertical (*Z*) dimension. Pooled data: *n* = 8 rats, 5 days, 2 trials per day and rat.

Cube crossings were calculated from rats’ paths in a similar way as for bin-crossings in Experiment 1. Animals exhibited significantly more horizontal movements (*X*: 46.23 ± 5.16, *Y*: 50.34 ± 5.75 cube-crossings) than vertical movements (*Z*: 9.97 ± 0.63) (*F*_2,27_ = 24.65, *P* < 0.001, Bonferroni tests: *X*–*Y*, *P* > 0.05; *X*–*Z*, *P* < 0.001; *Y*–*Z*, *P* < 0.001, see Fig. 7). To rule out that these results were simply an effect of the experimental design, horizontal and vertical movements of the optimised data sets were calculated. No significant differences between movements (horizontal: *X* 8.28 ± 0.34, *Y* 8.65 ± 0.34 and vertical: *Z* 8.24 ± 0.37) were observed (*F*_2,21_ = 2.85, *P* > 0.05, Bonferroni tests: *X*–*Y*, *P* = 0.19, *X*–*Z*, *P* > 0.20, *Y*–*Z*, *P* = 0.13).

**Fig. 8:**
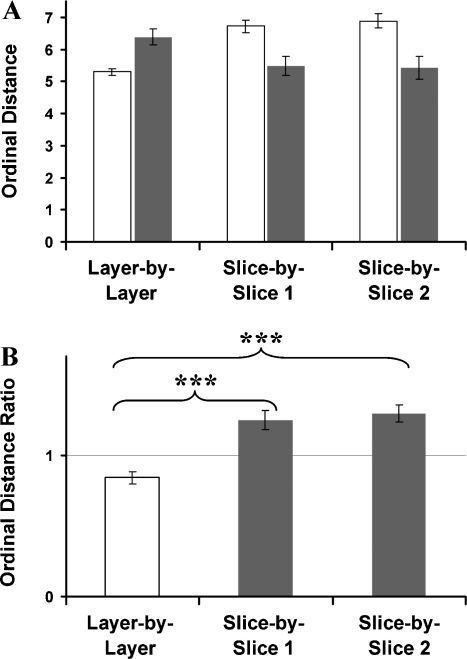
Experiment 3—(A) the ordinal distances of the foraging experiment on the lattice maze are shown for rat data (white) and optimised data (grey). (B) Ordinal distance ratios for the layer-by-layer analysis (white) and both slice-by-slice analysis (grey). The ordinal distance analysis was undertaken to underpin regularities in the food retrieval pattern on a given trial. Ordinal distance ratios were calculated by dividing ordinal distances of rats with ordinal distances of optimised paths. Smaller values in the layer analysis indicate clustering of choices within layers and therefore indicate that animals would be biased towards adopting a layer strategy, and higher values would indicate that animals used a vertically biased strategy. Pooled data: *n* = 8 rats, 5 days, 2 trials per day and rat.

Ordinal distances were calculated for the data sets (Fig. 8A). For real data, the ordinal distance obtained by the layer (*X*–*Y*) analysis was 5.30 ± 0.11 and for the slice (*X*–*Z* and *Y*–*Z*) analysis 6.72 ± 0.19 and 6.89 ± 0.23. Corresponding analysis of optimised data revealed a value of 6.38 ± 0.25 for the layer analysis and 5.48 ± 0.28 and 5.42 ± 0.35 for the slice analyses. A two-factor ANOVA showed a main effect of data type (real data vs. optimised data, *F*_1,42_ = 7.22, *P* < 0.05). No main effect was present when comparing dimensions (layer vs. slice, *F*_2,42_ = 0.94, *P* > 0.05). However there was a significant interaction between data type and dimension (*F*_2,42_ = 16.45, *P* < 0.001).

To extend the analysis, and to compensate for the unequal numbers of layers and slices, ordinal distance ratios were calculated as in Experiment 1 to analyse whether rats moved randomly around the maze or searched layer by layer. There were significant differences between the three spatial dimensions (*F*_2,21_ = 18.83, *P* < 0.001, Fig. 8B) The layer analysis (*X*–*Y*: 0.84 ± 0.04) revealed significantly smaller ordinal distance ratios when compared with both slice analyses (*X*–*Z*: 1.25 ± 0.07, *Y*–*Z*: 1.29 ± 0.06; Bonferroni Post Hoc Tests: *X*–*Y* vs. *X*–*Z*, *P* < 0.001; *X*–*Y* vs. *Y*–*Z*, *P* < 0.001), while the two slice dimensions did not differ (Bonferroni Post Hoc Tests: *X*–*Z* vs. *Y*–*Z*, *P* > 0.2). This indicates that, as with the pegboard, rats were biased towards remaining in a given horizontal plane when foraging for food.

### 3.4 Experiment 4: detour task on the lattice maze

The detour task began, as on the pegboard, with a training period in which rats learned to shuttle back and forth between a lower and upper food position.

Unexpectedly, during training the animals did not use a direct straight path to reach the upper position, but navigated horizontally to the corner below the target region and then vertically upwards. Such a two-step navigation strategy was not present when navigating downwards from the upper to lower target locations; here, rats preferred direct paths to reach the goal.

To quantify these observations a solid barrier was placed into the maze which forced rats to detour around the obstacle, taking either a vertical-first or vertical-last route. As on the pegboard rats showed a very strong preference for the initially horizontal route when navigating upwards (59 initially horizontal paths out of 60 completed trials, one sample *t*-test, *t*_5_ = 56, *P* < 0.001). This result was not surprising as animals even during training preferred this route and did not choose the diagonal, more direct path. Neither of the two routes were chosen significantly more often during downward navigation (22 initially horizontal paths out of 60 completed trials, one sample *t*-test, *t*_5_ = 1.8, *P* = 0.13), although a trend for initial vertical movements was present.

## 4 Discussion

In this study, we aimed to find out how rats distribute their time between horizontal and vertical movements in foraging and detour paradigms, using both a planar and a volumetric maze. The study was motivated by our electrophysiological findings that place neurons encode vertical space more coarsely than horizontal space [6], together with the hypothesis advanced by Grobety and Schenk [5] that the vertical component of a place-learning task tends to be solved before the horizontal component. We expected anisotropic behaviour, but based on Grobety and Schenk's findings we had expected a preference for vertical movements. However, we found that the animals in fact generally showed a horizontal bias in their behaviour. The reasons for the difference between our results and those of Grobety and Schenk are not clear but may be to do with the different task requirements of goal-directed navigation (their task) vs. self-directed foraging and route choice (our tasks).

The preference for horizontal movements seen in the rats in our study was manifested in several different ways: they exhibited horizontal movement preferences (Experiments 1 and 3), horizontally layered foraging strategies (Experiments 1 and 3) and horizontal-first route preferences when offered two equivalent routes to a goal (Experiments 2 and 4). Taking the behavioural and neural results together, we propose that rats represent and use space anisotropically, with preference given to the horizontal dimension. The results and their implications are discussed in greater detail below.

### 4.1 Horizontal movement preferences

In both kinds of apparatus, during free movement, rats preferred to make horizontal movements rather than vertical ones (shown in Figs. 3 and 7). On the pegboard, horizontal movement distance exceeded vertical by a factor of 2.73 (week 1) and 2.46 (week 2), whereas in the lattice maze the ratio rose to 4.65 (*X* vs. *Z* dimension) and 5.07 (*Y* vs. *Z* dimension). These horizontal movement biases were not simply a result of the experimental design, as horizontal (*X* and *Y*) and vertical (*Z*) movements of the optimised routes were the same. Because locomotion consisted of stepping from peg to peg, there is no particular reason to think that it was physically more difficult to move horizontally than vertically, and indeed the animals proved quite adept at climbing straight up. However, since climbing requires more energy, the preference for horizontal movements may result from the increased energetic cost of climbing. An alternative explanation might be that rats prefer to move horizontally because this is the plane in which the sensory and locomotor organs are oriented, thus making both sensory perception and locomotion easier in the horizontal plane.

The results obtained from the detour experiment are less straightforward. On the pegboard, rats quickly learned to execute both the outwards and return trips in a straight line, thus going diagonally across the board. However, on the lattice maze rats did not take the shortest route during training when navigating from the lower to the upper reward point, though they did so on the reverse trip. Instead, all the animals, in the majority of trials, navigated horizontally to the opposite corner and then vertically up. One possible explanation is that this dog-legging is a manifestation of thigmotaxis—the reluctance of rats to venture away from the boundaries of an apparatus and into the more exposed central areas. This may explain why the effect did not occur on the pegboard, which entirely consists of a large wall. It is also supported by our observation that the animals tended to spend more time on the edge of the lattice and less in the centre (Experiment 3). However, it is not clear why thigmotaxis would operate when going up but not when going down, unless it is because rats cannot see below them so easily when climbing, and dislike more being exposed in that direction. An alternative possibility is that the dog-leg route is preferred when going up in the unconstrained apparatus for the same reason as this route was preferred when detouring around the barrier—that animals prefer to execute the easiest part of a journey first, as a way of minimising energy expenditure. This possibility is explored further, below.

### 4.2 Horizontally layered foraging strategies

A horizontal bias was also observed in the foraging tasks. Foraging is a self-organised behaviour that allows an animal to maximise its return (usually food) while minimising its cost (energy, time, distance etc.). For terrestrial animals, the vertical component of a foraging journey makes a large contribution to energy expenditure, and thus would be expected to play an important role in shaping foraging strategies. Whereas in horizontal environments a “travelling salesman” (distance-minimising) solution is optimal, when there is a vertical component this solution should no longer be preferred. In agreement with this expectation, we found that rats used horizontally organised strategies during foraging in both environments. This was reflected in the ordinal distance calculations, which determined to what extent food items in a given horizontal band (layer), vs. vertical band (slice), tended to be retrieved together: these calculations revealed a significant propensity for rewards in a given layer to be collected together, which was not present either in the same data sliced vertically, or in the optimised control data. As a result of this layered behaviour, the rats’ paths deviated significantly from the optimal shortest-distance path determined using the travelling salesman algorithm.

What could be the adaptive advantage of such a strategy? The obvious answer is energy: given that movements against gravity are energetically more costly, a layered foraging strategy allows for minimisation of the vertical:horizontal movement ratio and thus minimisation of energy expenditure. As discussed above, this strategy produced 2.5–5-fold smaller vertical distances than would have occurred if the animals had used a shortest-distance path. However, there is another potential benefit to layered spatial behaviour, and that is its effect on cognitive load. Navigating freely and isotropically in 3D space requires maintenance of a volumetric, 3D mental map of the environment, together with the ability to update self-localization estimates in three dimensions. Moreover, there are two more degrees of rotation in three dimensions (in two orthogonal vertical planes) which make tracking heading and position far more complex, partly because rotations in three dimensions interact, and are therefore order-dependent. Construction of a layered 2D map would be far less costly, in terms of neural computing power. Indeed, we have obtained evidence that place neurons (place and grid cells in the mammalian hippocampus and entorhinal cortex, respectively) encode horizontal space to a high degree of precision but encode vertical space only weakly, if at all [6]. Our proposal is therefore that rats, and possibly all animals, encode space in the form of a stack of two-dimensional maps, rather than as a fully volumetric, 3D space. Whether this layered encoding is a cause or consequence of the layered behaviour we saw in our study is a matter for future research.

Given the additional difficulty of navigating in vertical space, an animal planning a path has to undertake a complex cost-benefit analysis to decide how much further it would be prepared to travel horizontally, in order to avoid a given amount of vertical travel. It remains unknown how rats trade off horizontal against vertical distance—do they have an inbuilt (i.e., evolved) ratio preference, or has this trade-off been learned with experience?

### 4.3 Horizontal-first route preferences during detouring

As well as during unconstrained movement, a horizontal bias was also found in goal-directed behaviour. When two possible routes to reach a goal were presented on the pegboard, by introducing a symmetrically placed barrier around which rats had to detour, the animals quickly developed a preference for the route having a strong shallow-first component over that in which the initial leg was steep. On the lattice maze, as discussed earlier, rats preferred such a route on the upwards journey even before the barrier was placed in the maze. Insertion of the two types of barriers (Fig. 2D: solid barrier and Supplementary Fig.S5: 16-hole barrier) highlighted this effect, with rats using paths with a strong initial shallow component (solid barrier) and the lowest hole (16-hole barrier: hole 16) to reach the goal significantly more often.

Interestingly, on the pegboard, this preference for the shallow-first route was reversed on the very first trial, in which the overwhelming majority of rats preferred the steep-first route (Fig. 5). We speculate that this might be an anxiety response to the sudden change in maze conditions. However, attempts to induce a similar novelty/anxiety response by changing the colour of the environment did not provoke similar climbing behaviour (data not reported), and so it is not clear why such a clear climbing response was elicited. The first trial notwithstanding, rats thereafter overwhelmingly preferred the horizontal-first journey, even though both journeys were equivalent in terms of overall climbing. This finding is in line with human performances in a similar environment, whereby horizontal paths were executed first [2].

We suggest two possible explanations for this preference. First, rats faced with a choice of two paths might apply a simple heuristic, always choosing the segment that involves less effort first. Similar experiments in humans [1] have shown that when navigating in horizontal environments participants prefer using an “initial segment strategy”, meaning that when routes of equal length are presented, the ones with a longer initial segment are favoured [1,4]. Humans may assume that long initial segments are a characteristic of short routes [1]. Thus, it may be that humans and animals compare only first segments of a route, and based on this, draw assumptions on the whole route, in order to reduce cognitive load.

An alternative possibility is that rats show temporal discounting of effort—that is, rats may prefer to defer paying a cost (in this case, climbing), in a cost-benefit trade-off. While temporal discounting of reward (preferring small reward immediately to larger reward later) is a well-studied phenomenon, at least in humans, a similar phenomenon for effort has not yet been well studied, in humans *or* in rats. Temporal discounting is an adaptive heuristic that takes into account the growing uncertainty of later events. In the case of reward, the advantage of choosing the smaller reward sooner is that the later reward, although larger, may not occur at all. Likewise, in executing a journey with low-cost and high-cost subcomponents, if the journey might be interrupted (which is not unlikely for prey animals) then it is better to execute the low-cost subcomponents first, so as to have wasted as little effort as possible if interrupted. These two alternatives (impulsivity vs. temporal discounting) differ in the degree to which the animal has processed information about the later legs in the journey, and determining which is the case in rats is a matter for future study.

The preference for the horizontal-first journey was relative rather than absolute. When the barrier was offset, so that the two routes now differed in how long/direct they were, it was possible to enhance or reverse the animal's choices. We cannot say whether the relevant factor was the added length of the journey or the fact that that route would require a greater initial starting angular deviation from the goal vector. Studies in humans show a preference for routes in which the initial segment had a small angular deviation from the goal vector [1].

Although Grobety and Schenk [5] found the same horizontal movement biases as in our study, their place learning experiments indicate the opposite of our conclusion: that the vertical coordinates are learned first and that rats subsequently search for horizontal coordinates because of a preference for the vertical dimension. Their explanation for their findings was that since vertical movements require more energy they are more salient and so this dimension is learned more quickly. We have no good explanation as to why our findings were different, other than to note that we found that apparently irrelevant changes in apparatus, such as the transition from the 2D pegboard to the 3D lattice maze, caused alterations such as the dog-legging seen in the detour experiment. The implication is that a number of factors trade off against each other when rats are weighing up route choices. In general, however, there is agreement across all our experiments that rats prefer to execute the horizontal components of a journey first before undertaking the vertical, unless the efficiency gains achieved by combining them (as in the diagonal routes of the learning phase of the detour task) outweigh the other factors.

## 5 Conclusion

The present study has provided evidence that rodent navigation in 3D space involves strong horizontal biases, with rats using a “layer strategy” during foraging (i.e., preferring to complete foraging at a given height above ground before moving to a new level), and preferring routes to a goal in which the easier, shallow component of the route occurs first. We propose three contributing factors to these biases: (1) a layer strategy minimises vertical, high-energy movements; (2) rats prefer to delay climbing as long as possible, potentially a manifestation of temporal discounting of effort; and (3) because the neural encoding of height is coarse and low in information, organising movements around the horizontal plane is computationally less costly.

## Figures and Tables

**Table 1:** Experimental 2 × 2 design: the foraging and detour tasks were accomplished in two different environments, the pegboard and the lattice maze.

Environment	Task	
	Foraging	Detour
Pegboard	Experiment 1	Experiment 2
Lattice maze	Experiment 3	Experiment 4
