# Supplementary material for: Horizontal biases in rats’ use of three-dimensional space
Source: Behav Brain Res. 2011 Sep 23;222(2):279–88. doi: 10.1016/j.bbr.2011.02.035 (PMC3157560; doi:10.1016/j.bbr.2011.02.035)
Supplement: Supplementary file 1 [file mmc1.doc]

## Supplementary information

### Experiment 1: Foraging on the lattice maze

All rats rapidly acquired knowledge of the maze and became faster and more efficient in retrieving food within the maze. Both retrieval time and path length significantly decreased over time (Fig. S1), from 318 ± 20 seconds and 24 ± 2 meters on day 1 to 90 ± 5 seconds and 10 ± 1 meters on day 10 (retrieval time: F_9,135_ = 31.09, P < 0.001; path length: F_9,135_ = 19.07, P < 0.001).

**A**

**B**

*******

*******

**Figure S1:** Experiment 1 – Acquisition of foraging behaviour on the pegboard, showing mean (± SEM) latencies (A) and path lengths (B) to collect 16 food rewards. Pooled data: n = 8 rats, 10 days, 2 trials per day and rat.

Dwell times were compared across the five layers, and revealed a significant tendency for rats to prefer the topmost layer when the maze was relatively novel (Fig. S2). This was shown statistically as a significant effect of layers (*F*_4,45_ = 4.55, *P* < 0.01). Pairwise comparison using a Bonferroni correction revealed that rats spent significantly more time at the top layer (see table S1).

**Figure S2:** Experiment 1 – Dwell time on layers and movement behaviours during foraging on the pegboard. Bottom layer = layer 1 and top layer = layer 5. Mean (±SEM) dwell times.

**Table S1:** Dwell times in percent (Mean ± SEM) on layer 1 (bottom) – layer 5 (top) on the pegboard during foraging. Pairwise comparisons using a Bonferroni correction show that rats spent significantly more time at layer 5 (top).

| Layer | Mean ± SEM | Comparison of Layer 5 with other layers (1-4) |
| --- | --- | --- |
| 1 | 17.25 ± 0.86 | P < 0.05 |
| 2 | 15.47 ± 0.66 | P < 0.01 |
| 3 | 19.37 ± 0.65 | P > 0.05 |
| 4 | 20.90 ± 0.80 | P > 0.05 |
| 5 | 27.00 ± 1.03 |  |

### Experiment 3: Foraging on the lattice maze

**Learning curves**

In foraging on the lattice maze, rats showed similar foraging patterns as on the pegboard. Foraging times and path length declined from 248 ± 31 seconds and 19.47 ± 2.70 meters to 112 ± 10 seconds and 10.40 ± 0.70 meters (retrieval time: F_9,63_ = 5.34, P < 0.001; path length: F_9,54_ = 6.83, P < 0.001)**.**

**A**

**B**

**Figure S3:** Experiment 3 – Acquisition of foraging behaviour on the lattice maze, showing mean (± SEM) latencies (A) and path lengths (B) to collect 16 food rewards. Pooled data: n = 8 rats, 5 days, 2 trials per day and rat.

**Dwell times**

We observed that rats seemed to spend quite a lot of time on the outer regions of the lattice maze, which seems surprising given that this area is more exposed. We therefore analysed dwell times. Analysis of dwell times revealed a significant effect of layers (F_3,16_ = 11.80, P < 0.001; Fig. S3) Pairwise comparison using a Bonferroni correction showed that, as with the pegboard, rats spent significantly more time at the top layer (see table S2).

**Figure S4:** Experiment 3 – Dwell time on layers and movement behaviours during foraging on the lattice maze. Bottom layer = layer 1 and top layer = layer 4. (A) Mean (±SEM) dwell times shown for the lattice maze. Pooled data: n = 8 rats, 5 days, 2 trials per day and rat.

**Table S2:** Dwell times in percent (Mean ± SEM) on layer 1 (bottom) – layer 4 (top) on the lattice maze during foraging. Pairwise comparisons using a Bonferroni correction show that rats spent significantly more time at layer 4 (top).

| Layer | Mean ± SEM | Comparison of Layer 4 with other layers (1-3) |
| --- | --- | --- |
| 1 | 22.01 ± 1.66 | P < 0.01 |
| 2 | 20.52 ± 1.47 | P < 0.01 |
| 3 | 20.12 ± 1.23 | P < 0.01 |
| 4 | 37.35 ± 1.87 |  |

For further analysis each layer was divided into an outer (12 outer bins) and an inner (4 middle bins) region. Average dwell times per bin of inner and outer regions were calculated and averaged across layers and sessions. This approach revealed that rats spent significantly more times in outer regions of each layer (outer region (%): 57.31 ± 1.65, inner region (%): 42.69 ± 1.65, paired t-test: t_7_ = 4.43, P < 0.01).

Thus, rats spent more time on the top layer of the maze, and more time on the outer sections of the maze. We speculate that this may be because they prefer the better view of the environment afforded by these positions.

### Experiment 4: Detour task on the lattice maze


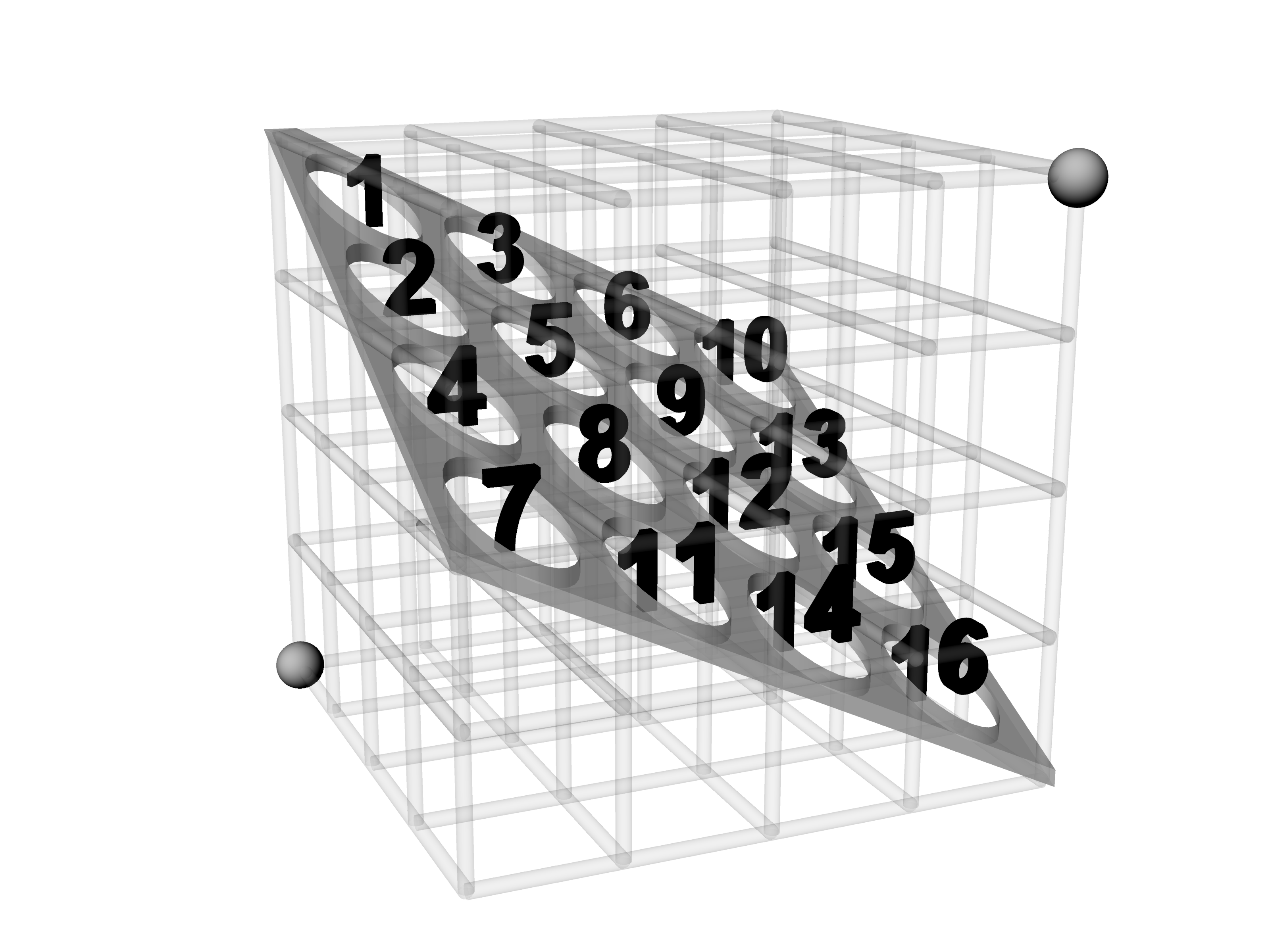


**UP**

**DOWN**

**B**

**C**


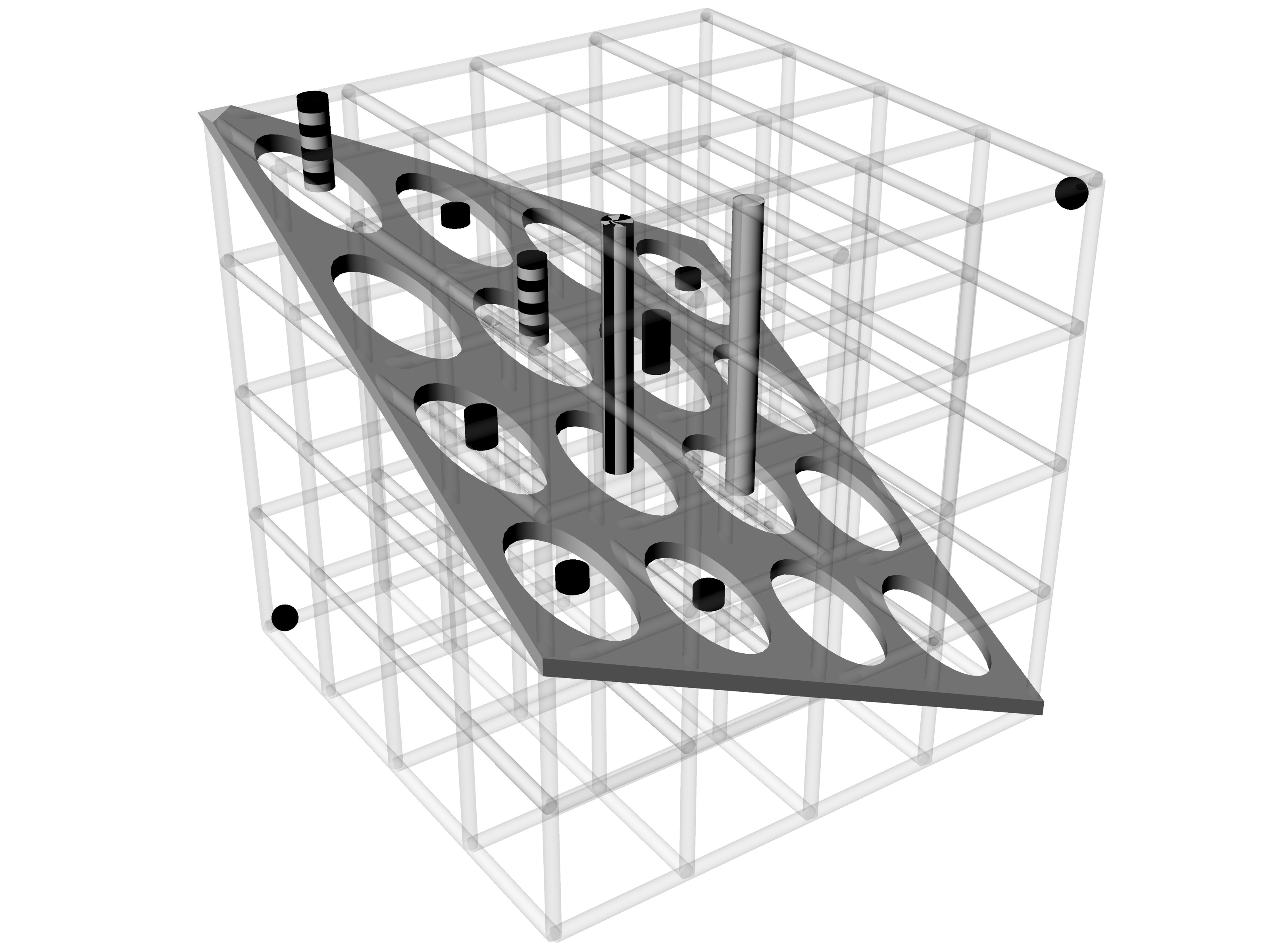

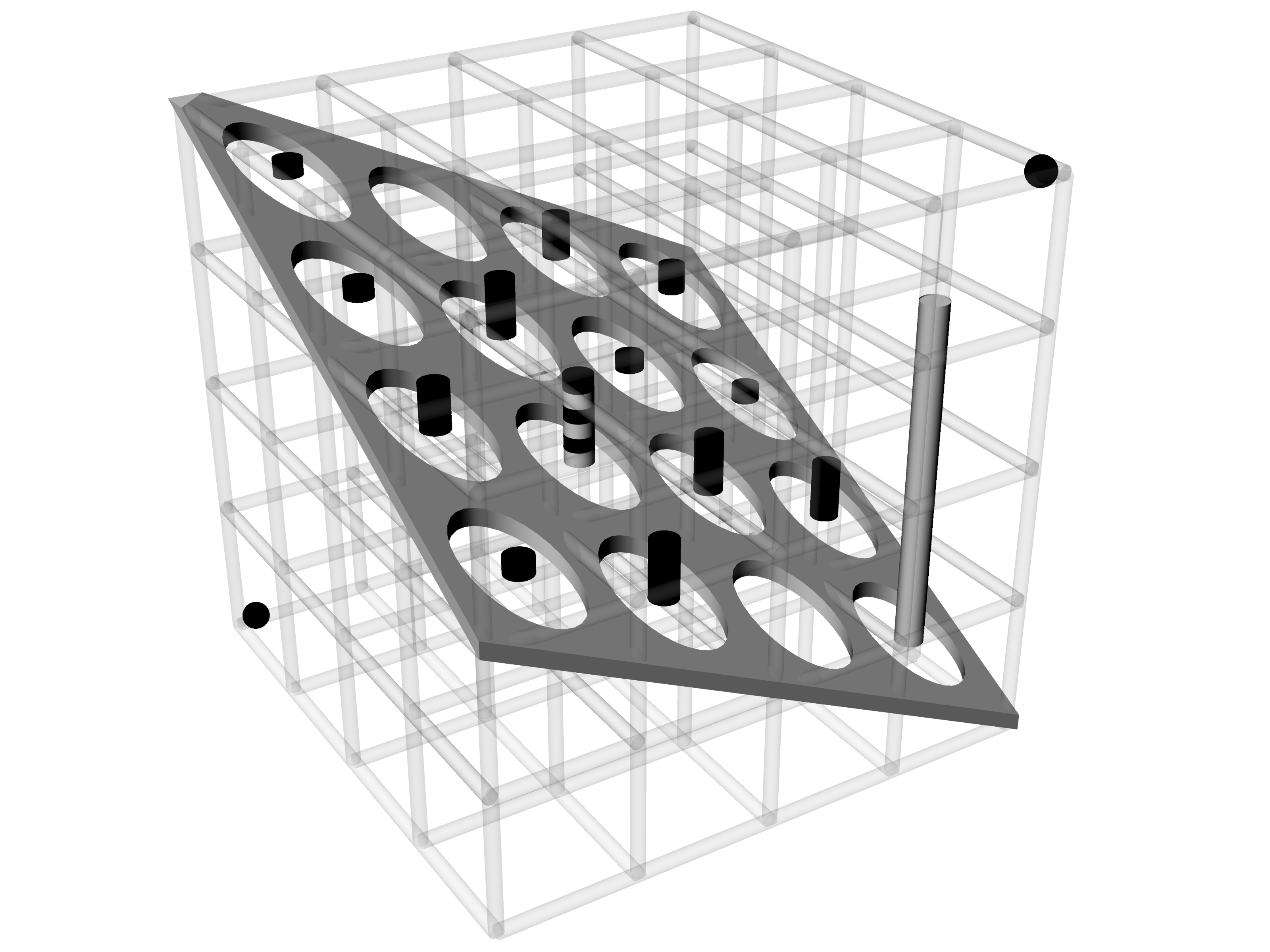


**1-5 11-15 21-25**

**6-10 16-20**

**Choice frequency**


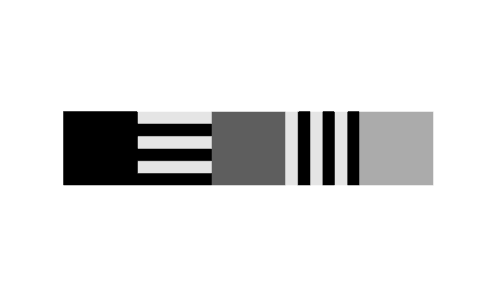


**A**

**Figure S5: Experiment 4 –** Schematic of the lattice maze detour experiment with a 16-hole barrier inserted is shown in (A). Path choices in the detour experiment on the lattice maze when navigating (B) upwards and (C) downwards. Bar heights and colours indicate the choice frequencies. Frequency rates were separated into five groups with a maximum frequency of 25. Group categories were as follows: 1-5 (black), 6-10 (stripes), 11-15 (dark grey), 16-20 (dots) and 21-25 (light grey). Pooled data: n = 6 rats, 10 trials per rat.

The lattice maze is shown with the inserted 16-hole barrier as it was positioned during testing. Start and goal positions are marked with the black spheres. Hole numbers indicate possible routes to cross through the barrier.

In order to highlight the results of the solid barrier a 16 hole-barrier (see Fig. S4) was introduced. The barrier was perforated with rat-sized holes to force the rats to choose their preferred path, and to make it easier for the experimenters to quantify the animals’ choice. Hole choices were recorded manually. As shown in figure, rats did not use the holes equally (upward navigation χ^2^_15_ = 140.53, P < 0.001; downward navigation: χ^2^_15_ = 160.27, P < 0.001).

Further analysis revealed that rats used, significantly more often, holes in the lower part of the barrier (lower holes 11-16: 38 times) than holes in the upper part (upper holes 1-6: 12 times) (binomial test lower holes vs. upper holes, P < 0.001). In particular, rats used the hole below the upper goal (hole 16) significantly more often than chance when navigating upwards (hole 16: 25/60 times; χ^2^_2_ = 128.44, P < 0.001). Thus, when going upwards, rats tended to use horizontal-first paths, as they had on the pegboard. No such differences were present during downward navigation (lower holes 11-16: 22 times, upper holes 1-6: 15 times; binomial test, P > 0.05). Indeed the two middle holes (8 and 12) were used significantly more often than expected by chance (hole 8: 17 times; χ^2^_2_ = 49.99, P < 0.001; hole 12: 21 times; χ^2^_2_ = 84.64, P < 0.001), indicating that rats chose direct paths towards the goal. Consequently the 16 hole-barrier revealed that rats did not use all holes equally: in upward navigation rats preferred navigating horizontally first, but when navigating downward preferred direct paths to reach the goal.
